# Use of peripheral vascular access in the prehospital setting: is there room for improvement?

**DOI:** 10.1186/s12873-020-00340-z

**Published:** 2020-06-09

**Authors:** Erin Gonvers, Thierry Spichiger, Eric Albrecht, Fabrice Dami

**Affiliations:** 1 Faculty of Biology and Medicine, University of Lausanne (UNIL), Lausanne, Switzerland; 2 Paramedic, Riviera Ambulances (ASR), La Tour-de-Peilz, Switzerland; 3 ES ASUR, Vocational Training College for Registered Paramedics and Emergency Care, Le Mont-sur-Lausanne, Switzerland; 4 Department of Anesthesiology, Lausanne University Hospital (CHUV), University of Lausanne (UNIL), Lausanne, Switzerland; 5 Department of Emergency Medicine, Lausanne University Hospital (CHUV), University of Lausanne (UNIL), Bugnon 46, 1011 Lausanne, Switzerland

**Keywords:** Peripheral vascular access, Prehospital, Out-of-hospital, Paramedic

## Abstract

**Background:**

Previous studies have shown that prehospital insertion of peripheral vascular access is highly variable. The aim of this study is to establish the proportion of peripheral vascular access placement and its use with regard to both the severity of cases and the main problem suspected by the paramedics involved. Over-triage was considered to have taken place where peripheral vascular access was placed but unused and these cases were specifically analysed in order to evaluate the possibility of improving current practice.

**Methods:**

This is a one-year (2017) retrospective study conducted throughout one State of Switzerland. Data were extracted from the state’s public health service database, collected electronically by paramedics on RescueNet® from Siemens. The following data were collected and analyzed: sex, age, main diagnosis suspected by paramedics and the National Advisory Committee for Aeronautics score (NACA) to classify the severity of cases.

**Results:**

A total of 33,055 missions were included, 29,309 (88.7%) with a low severity. A peripheral vascular access was placed in 8603 (26.0%) cases. Among those, 3948 (45.9%) were unused and 2626 (66.5%) of these patients had a low severity score. Opiates represent 48.3% of all medications given. The most frequent diagnosis among unused peripheral vascular access were: respiratory distress (12.7%), neurological deficit without coma or trauma (9.6%), cardiac condition with thoracic pain and without trauma or loss of consciousness (9.6%) and decreased general condition of the patient (8.5%).

**Conclusions:**

Peripheral vascular access was set in 26% of patients, nearly half of which were unused. To reduce over-triage, special attention should be dedicated to cases defined by EMS on site as low severity, as they do not require placement of a peripheral vascular access as a precautionary measure. Alternative routes, such as the intra-nasal route, should be promoted, particularly for analgesia, whose efficiency is well documented. Emergency medical services medical directors may also consider modifying protocols of acute clinical situations when data show that mandatory peripheral vascular access, in stroke cases for example, is almost never used.

## Background

The insertion of peripheral vascular access (PVA), intravenous (IV) or intraosseous (IO), is a common medical procedure in emergency medical services (EMS). However, there are no guidelines for its use in the prehospital setting and a lack of evidence supporting the efficacy of such a measure, especially in non-trauma or non-cardiac arrest patients.

Previous studies show that, once in place, prehospital-inserted PVAs were used in 17% [1] to 67% [2] of cases, with other PVAs being considered as a precautionary measure. The placement of PVA in the prehospital setting is motivated by the immediate need to give IV or IO medication or fluid therapy [3]. Paramedics are also taught to establish PVA as a precautionary measure because they might need to quickly administer medication or fluid, and inserting a catheter while the patient is stable might therefore be easier than in an emergency situation [4]. The circumstances in which a paramedic finds it appropriate to insert a catheter in the field are also determined by each individual, so a large amount of inconsistency exists [4].

Although it is a very rare occurrence, PVAs set by EMS were reported to be responsible for 28% of all cases of bacteraemia (mean dwell time 3.5 days) caused by PVAs inserted in the emergency department, wards and prehospital setting during a 5 year period [5]. The placement of a PVA should therefore be considered carefully before the insertion occurs, to avoid the “just-in-case” insertion and to promote procedures that truly benefit the patient. As hospitals propose guidelines toward greater efficiency with the Choosing Wisely® initiative [6], EMS’ should do the same and analyse their own practice to avoid unnecessary procedures and improve care recommendations.

The aim of this study is to establish the proportion of PVA setting and its use with regard to both the severity of the cases and the primary impression by the paramedics involved. Over-triage was considered to have taken place where PVAs were placed but unused. These cases were specifically analysed in order to evaluate the possibility of improving current practice.

## Method

This is a retrospective study conducted from January 1st 2017 to December 31st 2017 throughout the State of Vaud in the French-speaking region of Switzerland, where a centralised prehospital medical dispatch centre serves a population of 790,000 and handles over 80,000 calls per year.

This is a three-tier system. Paramedics use state protocols and algorithms for autonomous IV access (or IO when facing a life-threatening emergency and a complicated IV setting), cardiopulmonary resuscitation procedures, defibrillation and drug administration (crystalloids solutions, acetylsalicylic acid, adrenaline, amiodarone, clonazepam, diazepam, fentanyl, glucose, glucagon, isosorbide dinitrate, morphine, midazolam, naloxone, paracetamol, salbutamol, thiamine, and ondansetron). Paramedics are also allowed to use oral, subcutaneous, intra-rectal, intra-nasal, sublingual and intramuscular access. According to the state’s paramedic protocols [7], a precautionary or anticipated IV access is mandatory in case of childbirth and when a stroke is suspected. In other situations, the PVA is considered because of the need for systemic medications, for example in the case of anaphylaxis, pain with a visual analogue pain scale ≥3, haemodynamic instability, trauma, acute coronary syndrome, burn, seizure, cardiopulmonary arrest, suspected opioid intoxication, hypoglycaemia and the inability to give glucose orally. Paramedics can also ask for support from an emergency physician, either by telephone or on site, to administer other medications that are not included in their protocols.

With specific authorisation from the State’s public health services, data collected electronically on RescueNet® from Siemens by paramedics at the end of each mission were extracted.

The registry contains all ambulance protocols for intervention, including paediatrics protocols and patients who were not transported. Cases with a catheter inserted before EMS arrival, failure and/or refusal of insertion or missing data from the log were excluded, as well as inter-hospital transfers.

**Fig. 1:**
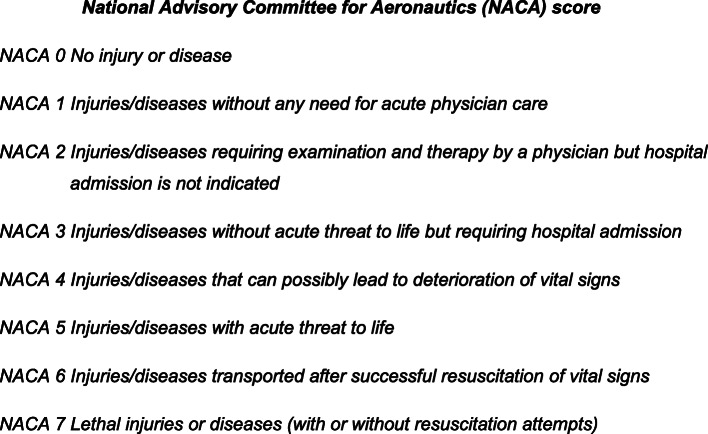
National Advisory Committee for Aeronautics (NACA) score

The following data were collected: sex, age, main diagnosis suspected by paramedics and the National Advisory Committee for Aeronautics (NACA) score (Fig. 1). The NACA score comprises eight categories ranging from 0 (no injury or disease) to 7 (lethal injuries or disease, with or without resuscitation attempts). It is determined by paramedics upon arrival at hospital and is defined by the most serious clinical state experienced at any given time during the mission. It does not rely on specific clinical or biological parameters but consists of classifying patients according to the most probable outcome of their current injuries or disease. The inter-rater reliability is considered acceptable [8]. It can efficiently discriminate between patients with regard to their short-term mortality [9]; a score ≥ 4 implies a potential life-threatening condition while a score < 4 may be classified as low severity [10]. The study team therefore decided to specifically analyse the latter category, as they may not need a PVA as a precautionary measure.

Details of any medication given through PVA were collected while information on medications given through intra-muscular, intra-nasal, intra-rectal and oral routes was not. The amount of vascular filling was taken into consideration (NaCl 0.9% and/or Ringer Lactate). Volumes equal to 500 mL or more were considered as a treatment for patients above 14 years of age, while 10 mL/kg was considered for patients under 14 years old. The national growth curve and the child’s sex and age were used to determine the weight [11].

Simple descriptive statistics were used to analyse population characteristics. All data were anonymised and entered into a computerised database (Microsoft Excel, Microsoft Corp., Redmond, Washington, USA). Categorical data are presented as counts and percentage frequencies.

## Results

A total of 35,088 primary missions were completed during the study period; of these, 2033 (5.8%) were excluded. In total, 33,055 missions were included, 8603 (26.0%) of which had a PVA inserted.

**Fig. 2:**
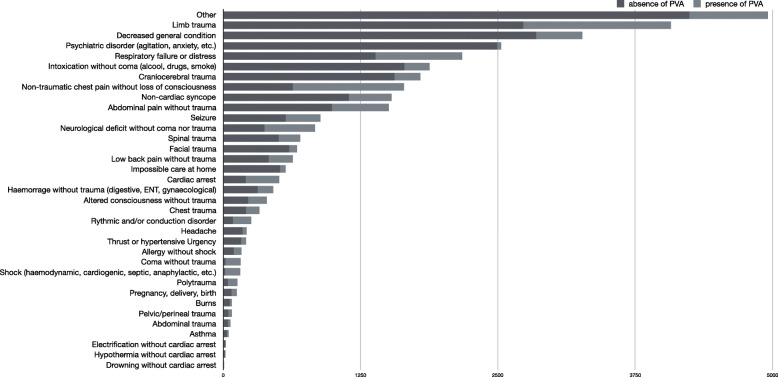
Count of the initiation of peripheral vascular access (PVA) by primary impression (case-mix)

**Fig. 3:**
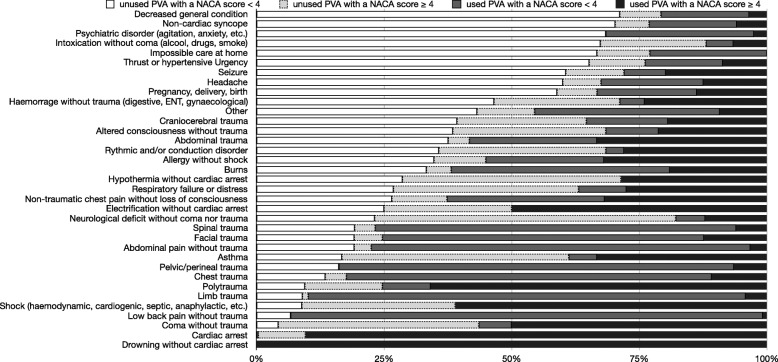
Percent of peripheral vascular access (PVA) used by primary impression and severity (NACA score)

Among patients with a NACA score < 4 (29,309; 88.7% of total), 5678 (19.4%) had a PVA, and among which 2626 (46.2%) PVAs were unused (Table 1). Among the unused PVAs, the most frequent diagnoses were: respiratory distress (12.7%), neurological deficit without coma or trauma (9.6%), non-traumatic chest pain without loss of consciousness (9.6%) and a decreased general condition of the patient (8.5%) (Table 2). (Supplementary file 1 details the primary impression of unused PVAs with a NACA score < 4 and Supplementary file 2 details the primary impression of unused PVAs with a NACA score ≥ 4). Figure 2 demonstrates the absolute number of PVAs placed by paramedic impression category (case mix of the study); the most frequent primary impressions have a very low proportion of PVA insertion (limb’ traumas, decreased general condition, and psychiatric disorders). Figure 3 demonstrates the percentage of PVAs used by impression category with regards to the severity of cases (NACA score); it allows primary impressions with a high proportion of unused PVAs and low severity (decreased general condition, non-cardiac syncope, intoxication without coma) to be differentiated from unused PVAs with high severity (neurological disorder without coma nor trauma).

**Table 1:** Patients’ characteristics

	Total	Patients without PVA	Patients with PVA	*Patients with used PVA*	*Patients with unused PVA*
Total *n (% of all patients)*	33′055 (100) [100]	24′452 (74.0) [100]	8′603 (26.0) [100]	4′655 (54.1)^§^ [100]	3′948 (45.9)^§^ [100]
Age *mean {*SD}	60,0 {25.5}	58.9 {26,5}	63.2 {22,2}	61.0 {22,6}	65.8 {21,6}
NACA score < 4 *n (%)*	29′309 [88.7]	23′631 (80.6) [96.6]	5′678 (19.4) [66.0]	3′052 (53.8)^§^ [65.6]	2′626 (46.2)^§^ [66.5]
NACA score ≥ 4 *n (%)*	3′746 [11.3]	821 (21.9) [3.4]	2′925 (78.1) [34.0]	1′603 (54.8)^§^ [34.4]	1′322 (45.2)^§^ [33.5]
Children ≤18 year old (%)	2′395 [7.2]	2′108 (88.0) [8.6]	287 (12.0) [3.3]	194 (67.6)^§^ [4.2]	93 (32.4) ^§^ [2.4]
Adult > 18 year old (%)	30′660 [92.8]	22′344 (72.9) [91.4]	8′316 (27.1) [96.6]	4′461 (53.6)^§^ [95.8]	3′855 (46.4)^§^ [97.6]

Abbreviations: *PVA* peripheral vascular acces; *NACA* National Advisory Committee for Aeronautics; *SD* Standard Deviation

[]: column percentages

(): row percentages

()^§^: row percentages related to patients with PVA only

**Table 2:** Primary impression of patients with an unused peripheral vascular access (PVA)

Number of cases	3948 (100)
Respiratory failure or distress	500 (12,7)
Neurological deficit without coma or trauma	380 (9,6)
Non-traumatic chest pain without loss of consciousness	378 (9,6)
Decreased general condition	335 (8,5)
Non-cardiac syncope	300 (7,6)
Seizure	228 (5,8)
Intoxication without coma (alcohol, drugs, smoke)	200 (5,1)
Craniocerebral trauma	153 (3,9)
Limb trauma	137 (3,5)
Altered consciousness without trauma	119 (3,0)
Abdominal pain without trauma	116 (2,9)
Rhythmic and/or conduction disorder	115 (2,9)
Haemorrhage without trauma (digestive, ENT, gynaecological)	101 (2,5)
Coma without trauma	61 (1,5)
Shock (haemodynamic, cardiogenic, septic, anaphylactic, etc.)	53 (1,3)
Spinal trauma	46 (1,2)
Impossible care at home	37 (0,9)
Thrust or hypertensive urgency	35 (0,9)
Pregnancy, delivery, birth	34 (0,9)
Allergy without shock	31 (0,8)
Cardiac arrest	29 (0,7)
Headache	27 (0,7)
Psychiatric disorder (agitation, anxiety, etc.)	26 (0,7)
Chest trauma	21 (0,5)
Polytrauma	21 (0,5)
Facial trauma	18 (0,5)
Low back pain without trauma	15 (0,4)
Asthma	11 (0,3)
Abdominal trauma	10 (0,2)
Burns	8 (0,2)
Hypothermia without cardiac arrest	5 (0,1)
Pelvic/perineal trauma	5 (0,1)
Electrification without cardiac arrest	4 (0,1)
Other	389 (9,8)

Among patients with a PVA, 4655 (54.1%) patients received at least one medication. The most frequent were: opiates (48.3%), acetylsalicylic acid (8.6%), and vascular filling (8.2%). (Supplementary file 3). The most frequent medications received by low severity patients (NACA < 4) were opiates (71.6%), anti-nausea treatment (10.3%) and acetylsalicylic acid (6.5%) (Supplementary file 4).

## Discussion

Only a small percentage of patients in this study received a PVA (26%), which is a significantly lower level than seen in other studies, where proportions varied from 50% [12] up to 60% [1]. Among those patients receiving a PVA, 46% were unused. This is lower than previous studies, where values of 83% [1] and 72% [13] were attributed to unused PVAs. This implies a small absolute number of unused PVAs globally. Nevertheless, as the insertion of a PVA is more complicated in the prehospital setting, especially regarding the asepsis procedure [14], the placement of such a device should be carefully assessed. EMS must consider many variables, including the clinical presentation of the patient, the paramedic’s differential diagnosis, EMS protocols, “gut feeling” and anticipation of a worsening scenario. The setting of a PVA is mainly validated in the case of a life-threatening emergency (principles of precaution) or when a medication or fluid is needed without an alternative route. EMS should not anticipate the use of a catheter by the hospital as hospital personnel would be able to place it under better conditions and perform a blood draw at the same time. It was shown that a PVA inserted in the prehospital setting but unused in the field did not shorten the time to accessing treatment once the patient arrived in the emergency department [15].

In this study, 66% of the unused PVAs were found to be among patients with a NACA score < 4. As these PVAs would not be considered precautionary in terms of case severity, they may be the easiest to avoid. Therefore, efforts to reduce over-triage should be centred on patients categorised by EMS as low severity. A NACA score < 4 is should not to be considered as an absolute reason not to not insert a PVA, but rather an invitation to reassess the need to perform such a procedure.

When looking specifically at prehospital diagnoses, some cases (intoxication without coma, neurological deficit without coma or trauma, impossible care at home, decreased general condition, hypertension, and hypothermia) had a level of unused PVAs of more than 50%. As some of these conditions required the placement of a PVA and were rated NACA ≥4, the decision to postpone the PVA placement must not be entrusted to paramedics. Medical directors should use these data to modify existing protocols where necessary. For example, in the case of a stroke registered as a “neurological deficit without trauma or coma” in this setting, the state’s protocol recommends the insertion of a PVA, but results showed that this represents 20.6% of unused PVAs among patients with a NACA score ≥ 4.

This study also showed that the prevailing medication with a NACA score < 4 was opiates, followed by anti-nausea treatment and acetylsalicylic acid. When dealing with low severity cases, it can be suggested that alternative routes could be used such as intramuscular injection in the case of seizure [16], or the intra-nasal route for analgesia. Regarding the latter, based on the published literature, the intranasal administration of fentanyl, sufentanil, ketamine, and hydromorphone may be a safe, effective, and well-tolerated alternative to intramuscular or intravenous administration in the prehospital and ED settings [17–19]. In this case-mix, if all patients with a NACA score < 4 who received opiates through PVAs (which represent 71% of patients in this category) had benefitted from an alternative route without a PVA being set, there would be a drop from 26 to 18% in the total number of patients with a PVA. This reflection process is probably best performed when dealing with children as the setting of a PVA is sometimes complicated in those cases; we observe a lower proportion of unused PVAs in this group of patients compared to adults (Table 1). Finally, with regard to acetylsalicylic acid, in the absence of impaired consciousness or difficulty swallowing, it has been validated to give it orally for patients presenting chest pain [20].

### Limitations

This is a retrospective study in a specific setting and may not be reproducible elsewhere; some data may have been lost or could be inaccurate resulting in misclassification, and medication may have been given but not documented. Transport time may have an impact on the decision to set a PVA, but these data were not available to the study team. Alternative routes of medication administration were not collected.

This study was not designed to estimate under-triage (situations where PVA was not placed but may have been a benefit to the patient) as we did not have access to the hospital charts, nor the failure to set a PVA.

This study was not specifically designed to measure how many PVA could have been avoided if alternative routes had been prioritised.

## Conclusion

Although only 26% of patients received a PVA in this study, nearly half were unused, which gives some room for improvement. To reduce over-triage, special attention should be dedicated to cases defined by EMS on site as low severity, as these cases do not require the placement of a PVA as a precautionary measure. Paramedics could also reduce the placement of PVAs if alternative routes, such as intramuscular and intra-nasal, were promoted, particularly for seizure and analgesia, whose efficiency is well documented. EMS medical directors may also modify protocols for acute clinical situations when data show that mandatory PVAs, in stroke cases for example, are almost never used.

## Supplementary information

**Supplementary file 1.** Prehospital diagnosis of patients with an unused peripheral vascular access and a NACA score < 4.

**Supplementary file 2.** Prehospital diagnosis of patients with unused peripheral vascular access and NACA score ≥ 4.

**Supplementary file 3.** Medication given (all patients).

**Supplementary file 4.** Medication given to patients with NACA score < 4.

## Data Availability

The data that support the findings of this study are available from the Health Service of the State of Vaud but restrictions apply to the availability of these data, which were used under license for the current study, and so are not publicly available. Data are, however, available from the authors upon reasonable request and with permission of the Health Service of the State of Vaud.

## Abbreviations

PVA: Peripheral vascular access
EMS: Emergency Medical Services
IV: Intravenous
IO: Intraosseous
NACA: National Advisory Committee for Aeronautics
